# Evaluation of Operating Room Staff Awareness of Environmental Sustainability and Medical Waste Management

**DOI:** 10.4274/TJAR.2024.231490

**Published:** 2024-09-17

**Authors:** Yekta Bektaş, Çiğdem Yıldırım Güçlü, Başak Ceyda Meço

**Affiliations:** 1 Ankara University Faculty of Medicine, Department of Anaesthesiology and Reanimation, Ankara, Turkey

**Keywords:** Anaesthetics, carbon footprint, environment, global warming, operating rooms, recycling

## Abstract

**Objective:**

This study aims to identify the obstacles to recycling and environmental sustainability habits in a university hospital’s operating room (OR) environment in Turkey and lay the groundwork for potential solutions.

**Methods:**

A questionnaire was used to measure current views among the 140 OR staff members aged 20-54 years. The survey assessed awareness and behaviors of recycling at home and in the OR, as well as awareness of environmentally safe anaesthesia practices.

**Results:**

Half of the participants believed that ORs significantly affected their carbon footprint, and most agreed that these environmental effects could be reduced. The primary barriers to recycling were inadequate knowledge, negative staff attitudes and insufficient services. Notably, 76% of participants paid attention to segregating OR waste, yet many lacked formal education about the environmental impact of their practices. Approximately 89% agreed that the environmental effects of ORs could be further reduced, with education being a critical need.

**Conclusion:**

The healthcare sector’s contribution to carbon emissions and waste production is significant, especially in ORs. The lack of education regarding ecological implications is concerning. Implementing standardized training programs and enhancing recycling services can substantially reduce the environmental impact of ORs, highlighting the need for a more sustainable healthcare system.

**Main Points**

• This study identified critical barriers in medical facilities that hinder the implementation of sustainable waste management, including inadequate staff training, employee disinterest and a lack of accessible recycling services.

• A pivotal finding is the absence of standardized environmental education in healthcare education. This underscores the urgent need for comprehensive and integrated training programs in medical schools.

• Enhanced environmental practices, especially in operating rooms, emerge as a potential area for improvement, focusing on the need for better education and recycling services.

• This research highlights the critical role of educational initiatives in environmental sustainability and emphasizes examining the actual conditions of medical workplaces to drive change.

• Comparisons with international studies illustrate common challenges in achieving environmental sustainability in healthcare, regardless of country or medical setting.

## Introduction

The increasing occurrence of global warming necessitates an urgent reassessment of environmental policies across all sectors, including healthcare. The healthcare industry, which is a significant contributor to carbon emissions, bears a substantial environmental footprint. Citing a University of Chicago study, the healthcare sector in the United States accounts for 8% of the nation’s total greenhouse gas emissions.^1^ Hospitals, with their energy consumption being 2-3 times higher than a residential building of comparable size, also generate approximately 5.9 tons of waste annually. Notably, operating rooms (ORs) are responsible for approximately 21% of this waste.^2^ Given that infectious waste requires specialized handling, such as incineration and chemical treatment, misclassification and improper management can exacerbate environmental damage.

This study explores current attitudes and practices concerning recycling and environmental sustainability within OR settings, along with a comparative analysis with prior studies to identify areas for improvement and understand how regional differences may impact these practices.

## Methods

This cross-sectional study was conducted at a single center, involving 140 participants from OR staff (including anaesthesiologists, surgeons, residents, nurses and cleaning staff) to medical students at Ankara University. Ethical committee approval was secured prior to the study from the Ankara University Faculty of Medicine, Human Research Ethics Committee (approval no.: İ4-165-19, date: 10.10.2019). Participants were provided survey forms and informed consent documents. Survey forms were recycled after digitization.

Our inclusion criteria were anyone who consented to participate in this study and of any age and gender who disposes of waste from the OR, consisting of anaesthesiologists, surgeons, residents, nurses, cleaning staff, and medical students. The criteria for exclusion were forms lacking essential demographic details like age, gender, and occupation, as well as forms where more than 20% of the survey questions were left unanswered. For additional information, refer to the study flowchart.

Before the analysis, we grouped occupations based on the observed waste generation amount and similar educational backgrounds into four main groups to simplify the analysis: Doctors (Anaesthesiologists/Surgeons), Nurses/Technicians (Anaesthesia Nurses/Technicians, OR Nurses), medical students, and cleaning staff.

Out of 18 questions we prepared for our survey (the full survey form is included in the Appendix 1), we questioned basic demographic information such as age, gender, occupation, and years the participant practiced. We also examined the following:

Awareness and behaviors related to recycling at home or OR, understanding of environmentally safe anaesthesia practices and behaviors.

Likert scales were used to assess participants’ beliefs about ORs’ environmental impact, the potential for minimizing this effect through waste management and energy consumption reduction, and willingness to alter their work practices to reduce these impacts.

We assigned five specific questions to those working in anaesthesia practice (anaesthesiologists and anaesthesia technicians). These questions inquired about whether sevoflurane or desflurane is safer for the environment, the ways in which these gases harm the environment, whether a low or high fresh gas flow is more environmentally safe, and whether participants have received training on the environmental effects of anaesthesia practice, including the source of this training. Additionally, we asked about the three most significant barriers they encountered in their work, their interest in receiving education about recycling in the OR, and an open-ended question regarding their desired changes in the OR to better protect the environment.

### Statistical Analysis

An initial sample size of 110 was selected randomly to assess the effect of sample size, and a power calculation was performed using G*Power 3.1.9.2. A minimum participant count of 60 was determined to be statistically significant with a 95% power and an α = 0.05 error probability.

The survey data were managed in a spreadsheet format and analyzed using SPSS v11.5. Continuous data were tested for normal distribution, and various statistical tests (t-test & Mann-Whitney U test for comparisons between two groups, ANOVA & Kruskal-Wallis variance analysis for comparing more than two groups) were employed to analyze the data. Pearson’s chi-square test or Fisher’s exact test was used to compare nominal variables. *P*=0.05 was accepted as the threshold for statistical significance.

## Results

Among the 140 participants, we excluded 8 participants with incomplete demographics and forms. The participants were between 20 and 54 years old, with a median age of 28, and the gender distribution was 66% female to 34% male (Table 1).

Fifty-six percent of the participants believed that ORs have an essential effect on the carbon footprint and global warming, whereas 44% expressed no opinion. Twenty-one percent said they frequently recycled at home, 30.3% sometimes did, and 24.2% rarely recycled at home (Table 2).

Eighty-nine percent of the participants agreed that OR environmental effects can be further decreased, and 73% stated that while working in the OR, they try to take measures to reduce the ecological impact of ORs.

Seventy-six percent of participants reported paying attention to segregating OR waste, whereas 14.6% rarely or never do. When asked about the preferred anaesthetic agent, most doctors (90%) and 64% of the anaesthesia technicians stated sevoflurane. Three-quarters of the participants knew sevoflurane was the safest anaesthetic agent compared with desflurane;^3, 4^ however, 27% stated they needed more information.

Forty-eight percent of the anaesthesia nurses/technicians believe that low-flow desflurane is the environmentally safest practice. At the same time, 75% of the physicians believe that low-flow sevoflurane is the safest, which we found statistically significant between the occupational groups (Table 3, *P *< 0.001).

Half of the doctors and 79.3% of the nurses/technicians expressed that they had no prior education about the effects of anaesthesia practices on the environment. Only 60% of the anaesthesia care providers had previous education on this topic. Within this group, 42% had information from a colleague, 18% from curricular sources, 9% from conferences, and 24 from other sources.

The most frequently reported barriers to OR recycling were inadequate knowledge (82.6%), negative staff attitudes toward recycling (75%), insufficient recycling services (44.6%), and time constraints (46.2%). Nearly all (95.5%) participants believed that education about OR recycling is necessary.

Based on the 63 responses, essential suggestions to lessen the environmental impact of ORs include prioritizing waste management and efficient handling of sterilization solutions, enhancing education and awareness through staff training and informative materials, improving OR infrastructure like ventilation systems, fostering a change in staff attitudes toward environmental practices and boosting operational efficiency by reducing workload and optimizing resource use such as electricity.

## Discussion

In recent years, environmentally safe medical and anaesthesia practices have gained increasing attention. Multiple organizations have published guides and statements highlighting the importance of minimizing the environmental impact of clinical practice and personal life. Most highlighted recommendations include the use of environmentally safer medications (local anaesthetics and nerve blocks being the safest option), equipment, ultra-low fresh gas rates when using inhaled agents, and reduction and reuse of materials, when possible, without compromising patient safety. Incorporating environmental education within the medical curriculum and emphasizing that conducting medical research itself can also increase the carbon footprint.^5, 6, 7^

Our study surveyed environmental awareness in a single center, revealing significant barriers to reducing the carbon footprint of ORs. Only half of the respondents recognized the ecological effects of ORs, and nearly all acknowledged the importance of recycling. Recognition of the importance of recycling is a positive indicator that healthcare professionals are willing to engage in sustainable practices. However, limited awareness of the broader ecological impacts of OR indicates the need for more comprehensive educational initiatives. Additionally, half of the participants cited time constraints, highlighting the need for changes that integrate sustainable practices into the workflow without adding to the workload. Providing dedicated staff and ensuring that sustainable practices are efficient and rationalizing waste segregation processes so that there are no uncertainties when managing or generating waste can help address these issues.

Although most respondents claimed to segregate OR waste, the need for proper education raises concerns about the effectiveness and safety of OR methods. Notably, 16% of patients were admitted to seldom segregating OR waste, highlighting the need for stringent waste segregation practices for infection control and health safety, especially in large hospitals with substantial waste generation.

Our study identified significant barriers to recycling in ORs, which is consistent with previous research. The most frequently reported obstacles were inadequate knowledge (82.6%), negative staff attitudes (75.0%), insufficient recycling services (44.6%) and time constraints (46.2%). Nearly all participants (95.5%) agreed that education about OR recycling is necessary. These findings were similar to those of McGain et al.^8^, where half of 780 fellows from the Australian and New Zealand College of Anesthetists reported inadequate recycling facilities as a primary barrier, alongside negative staff attitudes (17%) and inadequate information on recycling (16%). Similarly, Petre et al.^9^ found that while nearly all the 426 Canadian anaesthesiologists were willing to recycle at work, only 30% did so, citing a lack of support from hospital leadership (63%) and insufficient education (62%) as major barriers. The high willingness to recycle contrasted sharply with the low implementation rate, underlining the need for systemic support and appropriate educational initiatives.

Our study further revealed that only 35% of the participants had received any education on recycling, with a mere 21% having received formal education from the curriculum and conferences. This percentage compared to less than half (42.6%) of Petre et al.’s^9^ respondents who had received prior formal training. These educational gaps highlight the necessity for comprehensive and structured training programs to raise awareness and competence in sustainable practices.

Most of the nurses and technicians had no prior experience in their daily practices. While many agree that low fresh gas flow is safer, they consider low-flow desflurane to be the safer option for the environment compared with sevoflurane, avoiding high-impact anaesthetics such as desflurane and nitrous oxide is essential due to their substantial climate impact and limited clinical benefits.^5^

Moreover, our participants provided suggestions that they think minimize the environmental impact of ORs, including prioritizing waste management, enhancing education and awareness, improving OR infrastructure, fostering positive staff attitudes, and optimizing resource use such as electricity. These recommendations resonate with the current guidelines and reinforce the idea that multifaceted approaches are needed to address the environmental impact of ORs.^5, 6^

Incorporating environmental sustainability into formal anaesthesia education and research programs is vital. Anaesthesia providers should lead sustainability initiatives within healthcare organizations and collaborate with industry to enhance environmental practices. It is important that educational and policy initiatives must consider the realities of the OR environment, such as high patient turnover, and focus on practical, achievable training programs.

### Study Limitations

This study is limited by its single-center, small-scale nature, which may not represent the diversity of anaesthesiologists’ practices across Turkey. Non-response bias and acquiescence bias could also have influenced the results. Multi-center and more extensive scale studies are needed to gain a more comprehensive and accurate representation of workplace habits and barriers in Turkey.

## Conclusion

As the healthcare sector increasingly recognizes the environmental impact of inhalation agents, the current lack of education about their ecological implications has become a critical concern. In our study, most participants showed an interest in education, and nearly all expressed that they had yet to receive formal education on this issue. Standardized and repeatable curricula should be implemented in residency training programs; simulation-based programs can also help increase recycling awareness and behavior. With improved training and accessibility to recycling services, along with the widespread adoption of consistent recycling behaviors among OR staff, minor changes in daily practice can significantly reduce the impact of ORs on carbon emissions and waste production, fostering an eco-friendlier healthcare system.

**Click the link to access Appendix 1: **https://l24.im/HPGnih

## Ethics

**Ethics Committee Approval:** Ethical committee approval was secured prior to the study from the Ankara University Faculty of Medicine, Human Research Ethics Committee (approval no.: İ4-165-19, date: 10.10.2019).

**Informed Consent:** Participants were provided survey forms and informed consent documents.

## Supplementary Materials

Appendix 1 Click here to access: https://l24.im/HPGnih

## Figures and Tables

**Table 1. Demographic Data (n=132):** 

	**Median (years)**	**Range (years)**
Age	28	20-54
Years in practice	4	0.33-30
**Gender**	**Frequency (n)**	**Percentage (%)**
Female	87	65.9
Male	45	34.1
**Occupational group**	**Frequency (n)**	**Percentage (%)**
Doctors	46	34.8
Nurses/Technicians	45	34.1
Cleaning staff	15	11.4
Medical students	26	19.7

**Table 2. Do You Segregate Recyclable Waste at Home? (n=132):** 

	**Frequency (n)**	**Percentage (%)**
Always	9	6.8
Often	28	21.2
Sometimes	40	30.3
Rarely	32	24.2
Never	23	17.4

**Table 3. Which Anaesthesia Practice is Safe for the Environment? (n=55, P < 0.001):** 

		**Low-flow sevoflurane**	**High-flow sevoflurane**	**Low-flow desflurane**	**High-flow desflurane**	**No opinions**
**Doctors**	Frequency (n)	21	1	5	0	1
	Percentage (%)	75.0%	3.6%	17.9%	0.0%	3.6%
**Nurses/Technicians**	Frequency (n)	5	0	13	1	8
	Percentage (%)	18.5%	0.0%	48.1%	3.7%	29.6%
**Total**	Frequency (n)	26	1	18	1	9
	Percentage (%)	47.3%	1.8%	32.7%	1.8%	16.4%
